# Supplementary material for: Stress-induced vtRNA1-1 modulates redox homeostasis and ferroptosis susceptibility in hepatocellular carcinoma cells
Source: Redox Biol. 2026 Oct 13;98:104439. doi: 10.1016/j.redox.2026.104439 (PMC13693114; doi:10.1016/j.redox.2026.104439)
Supplement: Supplementary file 1 [file mmc1.pdf]

# Figure S1

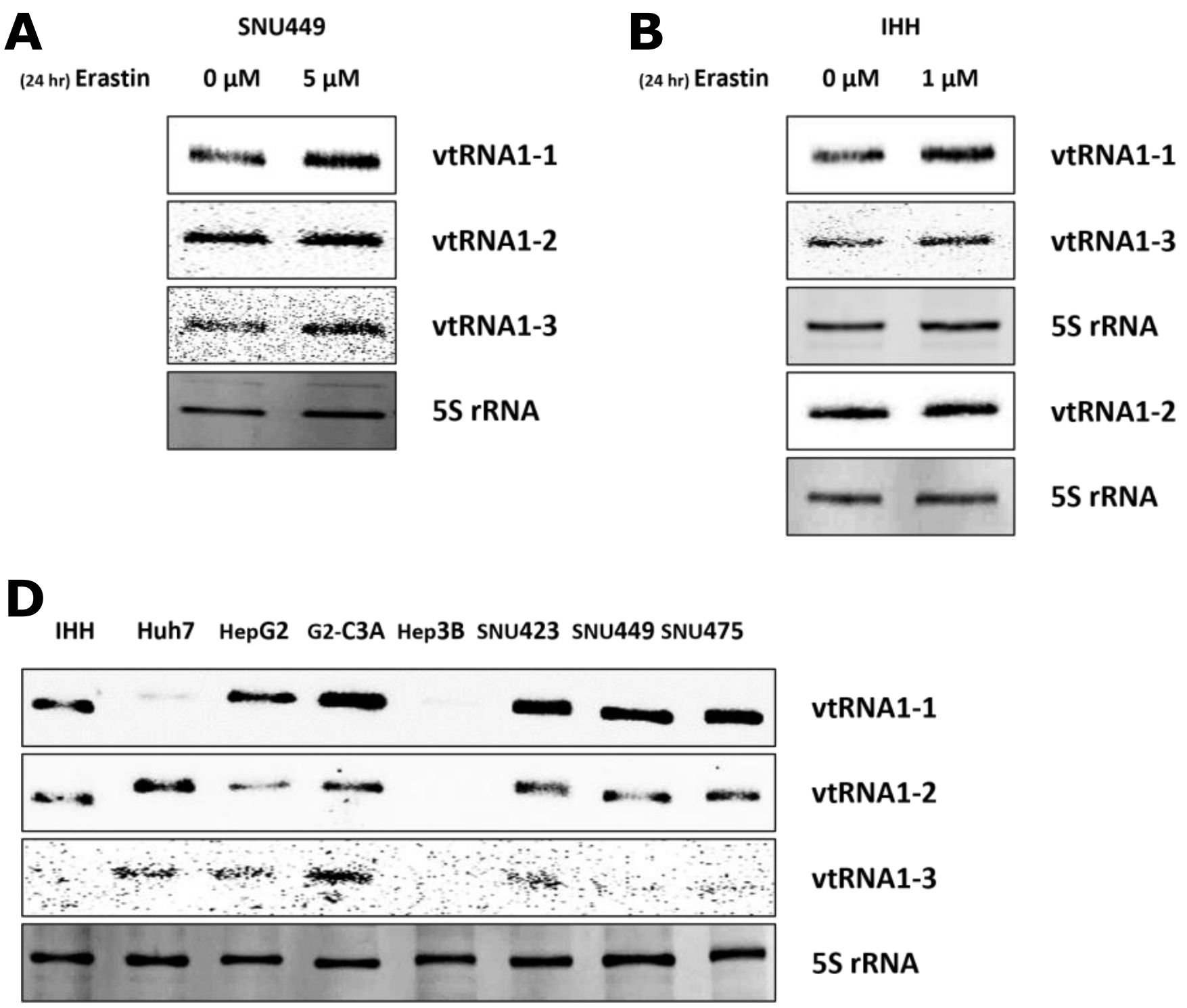


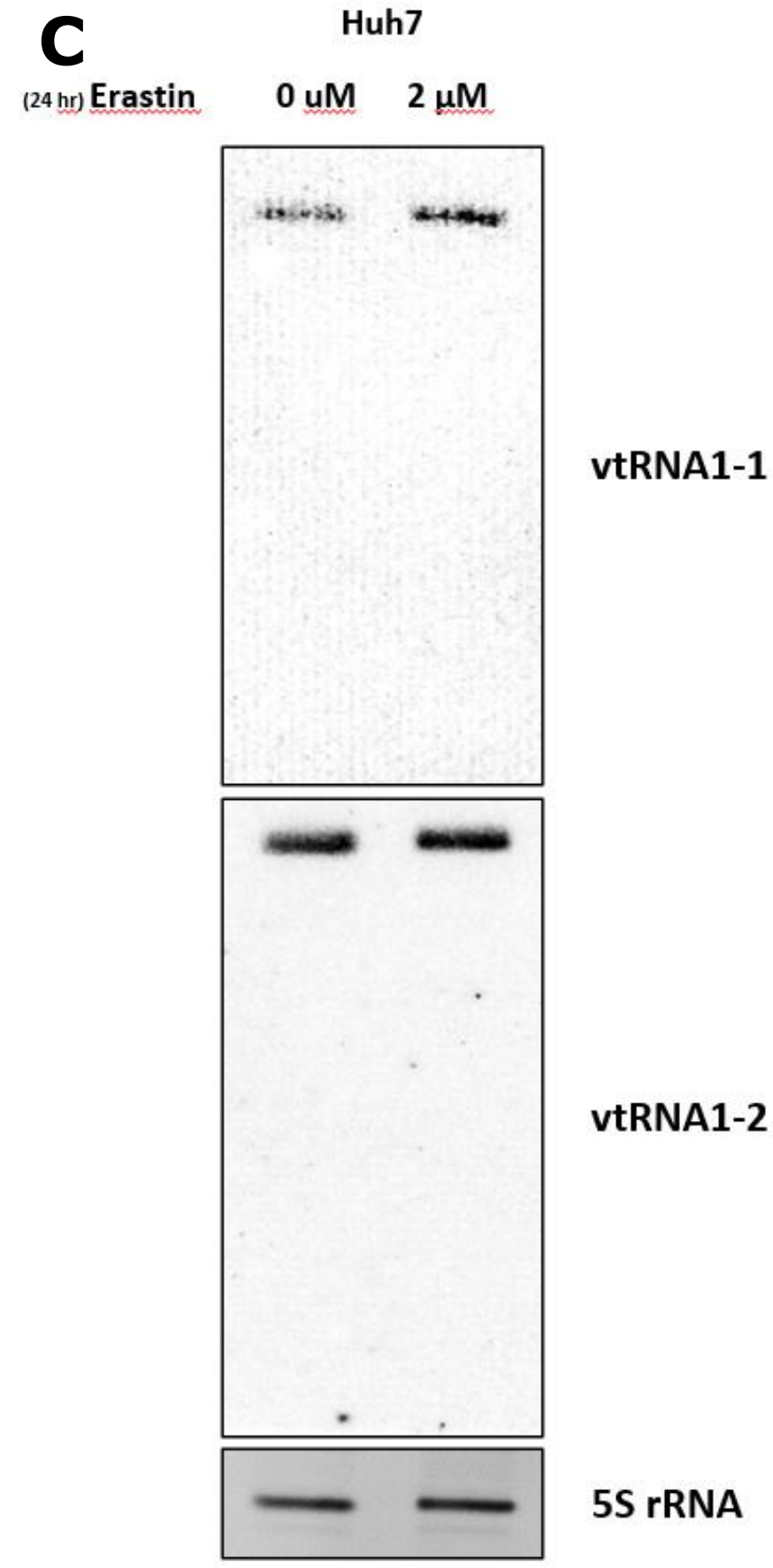


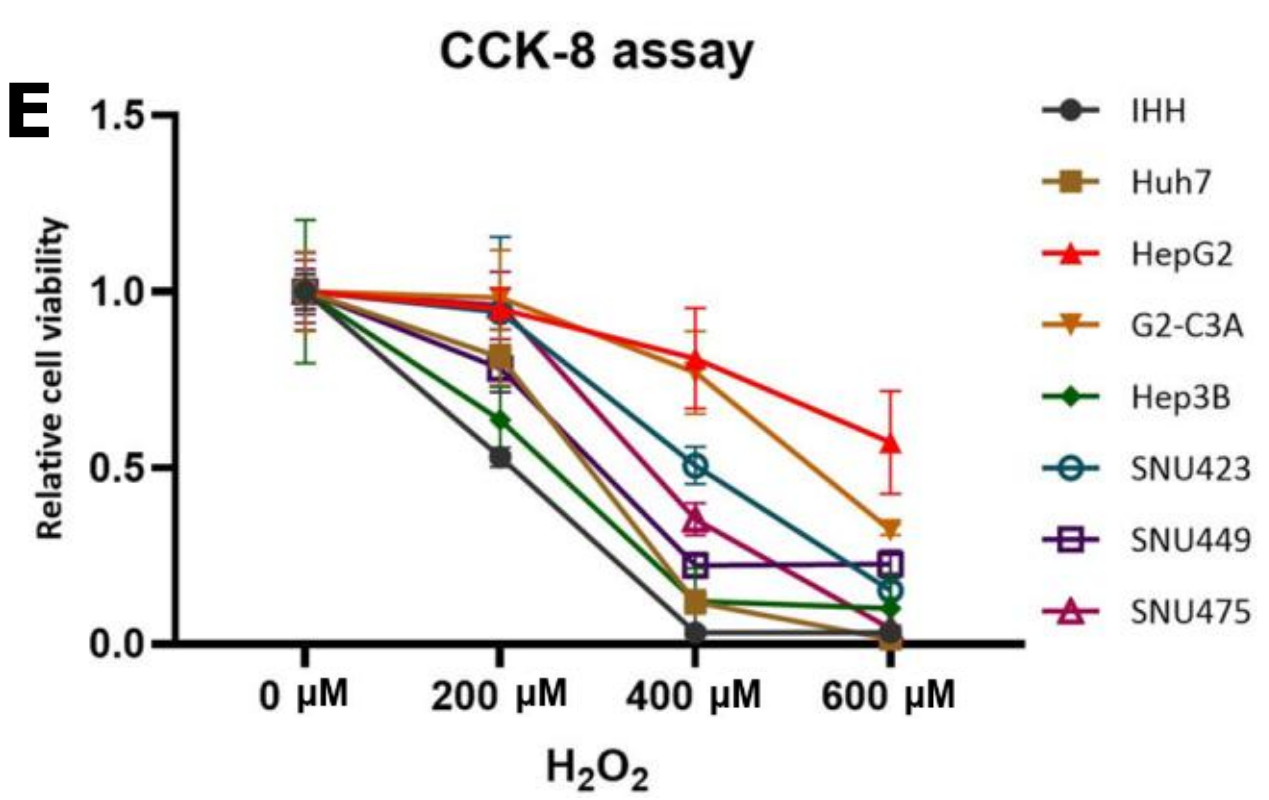


**F**

| | Huh7 | IHH | HepG2 | C3A | Hep3B | SNU423 | SNU449 | SNU475 |
|---|---|---|---|---|---|---|---|---|
| $H_2O_2$ (µM) | *284.92* | *194.20* | *845.25* | *509.90* | *239.86* | *381.02* | *307.61* | *343.46* |
| RSL3 (nM) | *61.52* | *62.01* | *302.16* | *198.54* | *48.26* | *107.03* | *234.26* | *197.30* |
| Erastin (µM) | *1.81* | *1.81* | *6.89* | *8.36* | *1.68* | - | - | - |

**Figure S1.** (A-C) Northern blot analyses of vtRNAs. Total RNA was extracted from indicated cells treated with erastin for 24 hours and analyzed. 5S rRNA served as a loading control. (D) Northern blot analysis of vtRNAs. Total RNA was extracted from eight human liver cell lines and analyzed. 5S rRNA served as a loading control. (E) The indicated cells were treated with $H_2O_2$ for 24 hours and the cell viability was measured by CCK-8 assay. (F) IC50 values of eight cell lines calculated based on Fig. 1E and S1E.

# Figure S2

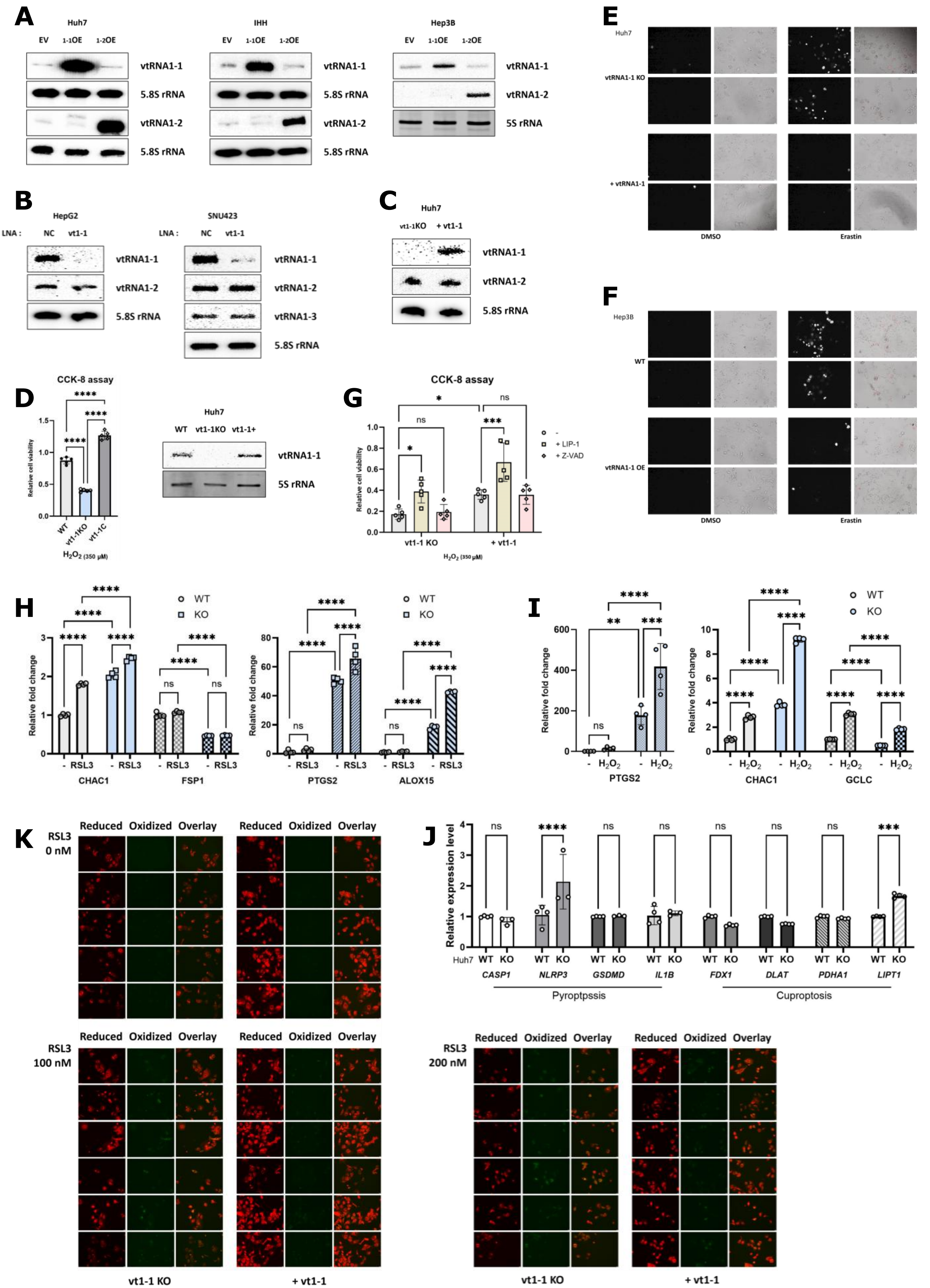
A
Huh7
EV 1-1OE 1-2OE
vtRNA1-1
5.8S rRNA
vtRNA1-2
5.8S rRNA
IHH
Hep3B
5S rRNA
B
HepG2
SNU423
LNA : NC vt1-1
vtRNA1-3
C
vt1-1KO + vt1-1
D
CCK-8 assay
Relative cell viability
WT vt1-1KO vt1-1C
H2O2 (350 μM)
WT vt1-1KO vt1-1+
E
vtRNA1-1 KO
+ vtRNA1-1
DMSO
Erastin
F
WT
vtRNA1-1 OE
G
+ LIP-1
+ Z-VAD
vt1-1 KO
+ vt1-1
ns
H
WT
KO
Relative fold change
RSL3
CHAC1
FSP1
PTGS2
ALOX15
I
GCLC
J
Relative expression level
Huh7
CASP1
NLRP3
GSDMD
IL1B
FDX1
DLAT
PDHA1
LIPT1
Pyroptpsis
Cuproptosis
K
RSL3 0 nM
RSL3 100 nM
RSL3 200 nM
Reduced Oxidized Overlay

**Figure S2.** (A and B) Northern blot analyses of vtRNAs. Following vtRNA1-1 or vtRNA1-2 overexpression (A) or LNA-mediated vtRNA1-1 knockdown (B), total RNA was extracted and analyzed. 5.8S rRNA and 5S rRNA served as loading controls. EV denotes empty vector and NC denotes negative control (mock transfection). (C) Northern blot analysis of vtRNAs. Following reintroduction of vtRNA1-1 into vtRNA1-1 KO Huh7 cells, total RNA was extracted and analyzed. 5.8S rRNA served as a loading control. (D) The indicated cells were treated with $H_2O_2$ for 24 hours, and the relative cell viability was measured by CCK-8 assay (left), normalized to the untreated cells. Northern blot analysis of vtRNA1-1. Total RNA was extracted from the indicated cells and analyzed. 5S rRNA served as a loading control. (right) (E and F) The indicated cells were treated with Erastin for 24 hours, and death cells were detected using propidium iodide staining. (G) The indicated cells were treated with $H_2O_2$ and indicated inhibitors for 24 hours, and the relative cell viability was measured by CCK-8 assay, normalized to the untreated cells. (H and I) qPCR analyses of the indicated mRNAs in Huh7 WT and vtRNA1-1 KO cells treated with RSL3 (H) or $H_2O_2$ (I) (n=4). (J) qPCR analysis of the indicated pyroptosis or cuproptosis markers in Huh7 WT and vtRNA1-1 KO cells (K) Lipid peroxidation was measured using C11-BODIPY fluorescent probe. After pre-treatment with RSL3, vtRNA1-1 KO and vtRNA1-1-expressing (vtRNA1-1 +) Huh7 cells were incubated with 10 μM of BODIPY 581/591 for 30 minutes in the dark. Images were captured using a fluorescence microscope. ****$p<0.0001$, *** $p<0.001$, **$p<0.01$, ns: non-significant (two-way ANOVA test)

# Figure S3

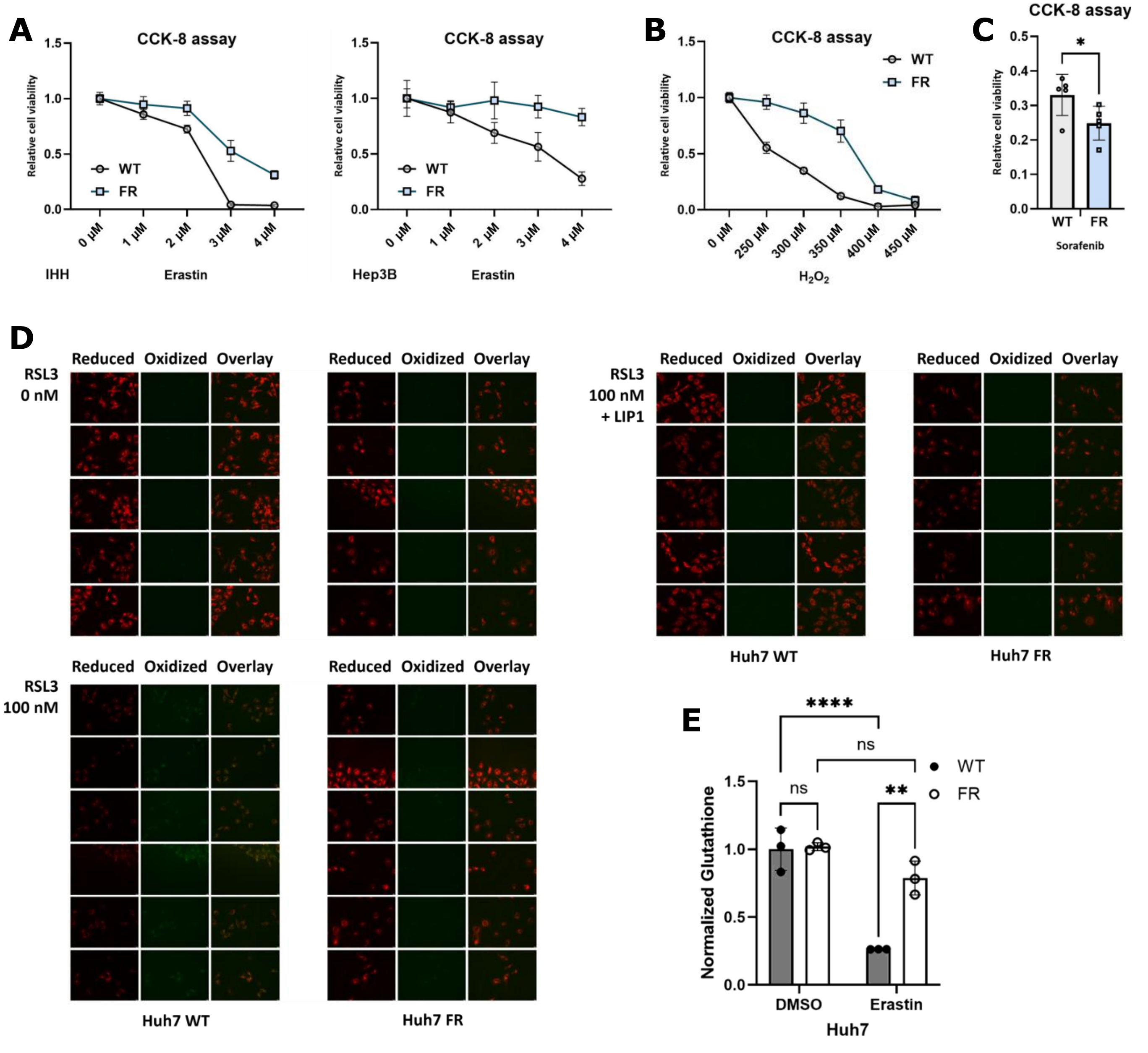

A
CCK-8 assay
Relative cell viability
WT
FR
0 μM
1 μM
2 μM
3 μM
4 μM
IHH
Erastin
CCK-8 assay
Relative cell viability
WT
FR
Hep3B
Erastin
B
CCK-8 assay
WT
FR
Relative cell viability
0 μM
250 μM
300 μM
350 μM
400 μM
450 μM
$H_2O_2$
C
CCK-8 assay
Relative cell viability
*
WT
FR
Sorafenib
D
Reduced
Oxidized
Overlay
RSL3
0 nM
RSL3
100 nM
+ LIP1
Huh7 WT
Huh7 FR
RSL3
100 nM
Huh7 WT
Huh7 FR
E
****
ns
ns
**
WT
FR
Normalized Glutathione
DMSO
Erastin
Huh7

**Figure S3.** (A-C) The indicated cells were treated with erastin (A), $H_2O_2$ (B) or sorafenib (C) for 24 hours and the cell viability was measured by CCK-8 assay. *$p<0.05$ (t-test) (D) Lipid peroxidation was measured using C11-BODIPY fluorescent probe. After pre-treatment with RSL3 and Lip-1, Huh7 WT and ferroptosis-resistant (FR) cells were incubated with 10 μM of BODIPY 581/591 for 30 minutes in the dark. Images were captured using a fluorescence microscope. (E) The indicated Huh7 cells were treated with Erastin for 16 hours, and total glutathione levels were measured.

# Figure S4

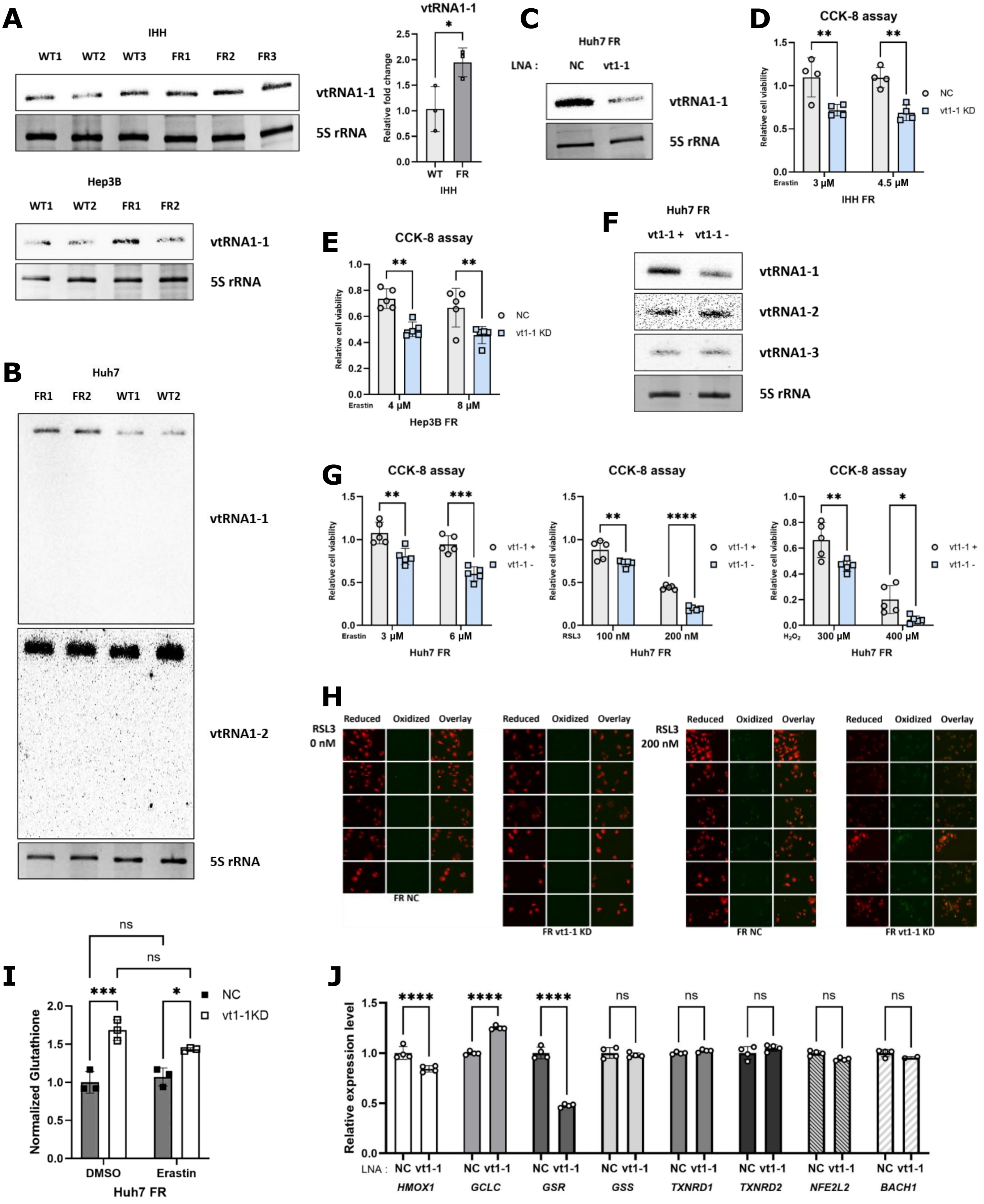

A
IHH
WT1 WT2 WT3 FR1 FR2 FR3
vtRNA1-1
5S rRNA
vtRNA1-1
Relative fold change
WT FR
IHH
Hep3B
WT1 WT2 FR1 FR2
C
Huh7 FR
LNA : NC vt1-1
D
CCK-8 assay
Relative cell viability
NC
vt1-1 KD
Erastin 3 μM 4.5 μM
IHH FR
E
Erastin 4 μM 8 μM
Hep3B FR
F
vt1-1 + vt1-1 -
vtRNA1-2
vtRNA1-3
B
Huh7
FR1 FR2 WT1 WT2
G
Erastin 3 μM 6 μM
RSL3 100 nM 200 nM
H₂O₂ 300 μM 400 μM
Huh7 FR
H
Reduced Oxidized Overlay
RSL3 0 nM
RSL3 200 nM
FR NC
FR vt1-1 KD
I
Normalized Glutathione
NC
vt1-1KD
DMSO Erastin
J
Relative expression level
LNA : NC vt1-1
HMOX1 GCLC GSR GSS TXNRD1 TXNRD2 NFE2L2 BACH1

**Figure S4.** (A and B) Northern blot analysis of vtRNAs. Total RNA was extracted from WT and FR cells and analyzed. 5S rRNA served as a loading control. The intensity of vtRNA1-1 in IHH was normalized to 5S rRNA (A, left, n=3). Error bars indicate the standard deviation. *$p<0.05$ (t-test) (C) Northern blot analysis of vtRNAs. Following LNA-mediated vtRNA1-1 knockdown, total RNA was extracted and analyzed. 5S rRNA served as loading controls. NC denotes negative control (mock transfection). (D and E) Following LNA-mediated knockdown of vtRNA1-1, indicated FR cells were treated with erastin for 24 hours, and the cell viability was measured by CCK-8 assay, normalized to the untreated cells. (F) Northern blot analysis of vtRNAs. Total RNA was extracted from control and CRISPR/Cas9-mediated vtRNA1-1-depleted FR cells and analyzed. 5S rRNA served as a loading control. (G) Following CRISPR/Cas9-mediated depletion of vtRNA1-1, Huh7 FR cells were treated with ferroptosis inducer for 24 hours, and the cell viability was measured by CCK-8 assay, normalized to the untreated cells. (H) Lipid peroxidation was measured using C11-BODIPY fluorescent probe. After pre-treatment with RSL3, control and LNA-mediated vtRNA1-1-depleted Huh7 FR cells were incubated with 10 μM of BODIPY 581/591 for 30 minutes in the dark. Images were captured using a fluorescence microscope. ****$p<0.0001$, *** $p<0.001$, **$p<0.01$, *$p<0.05$, ns: non-significant (two-way ANOVA test) (I) Following LNA-mediated knockdown of vtRNA1-1, indicated FR cells were treated with Erastin for 16 hours, and total glutathione levels were measured. (J) qPCR analyses of the indicated mRNAs in control and vtRNA1-1-knockdown Huh7 FR cells.

# Figure S5

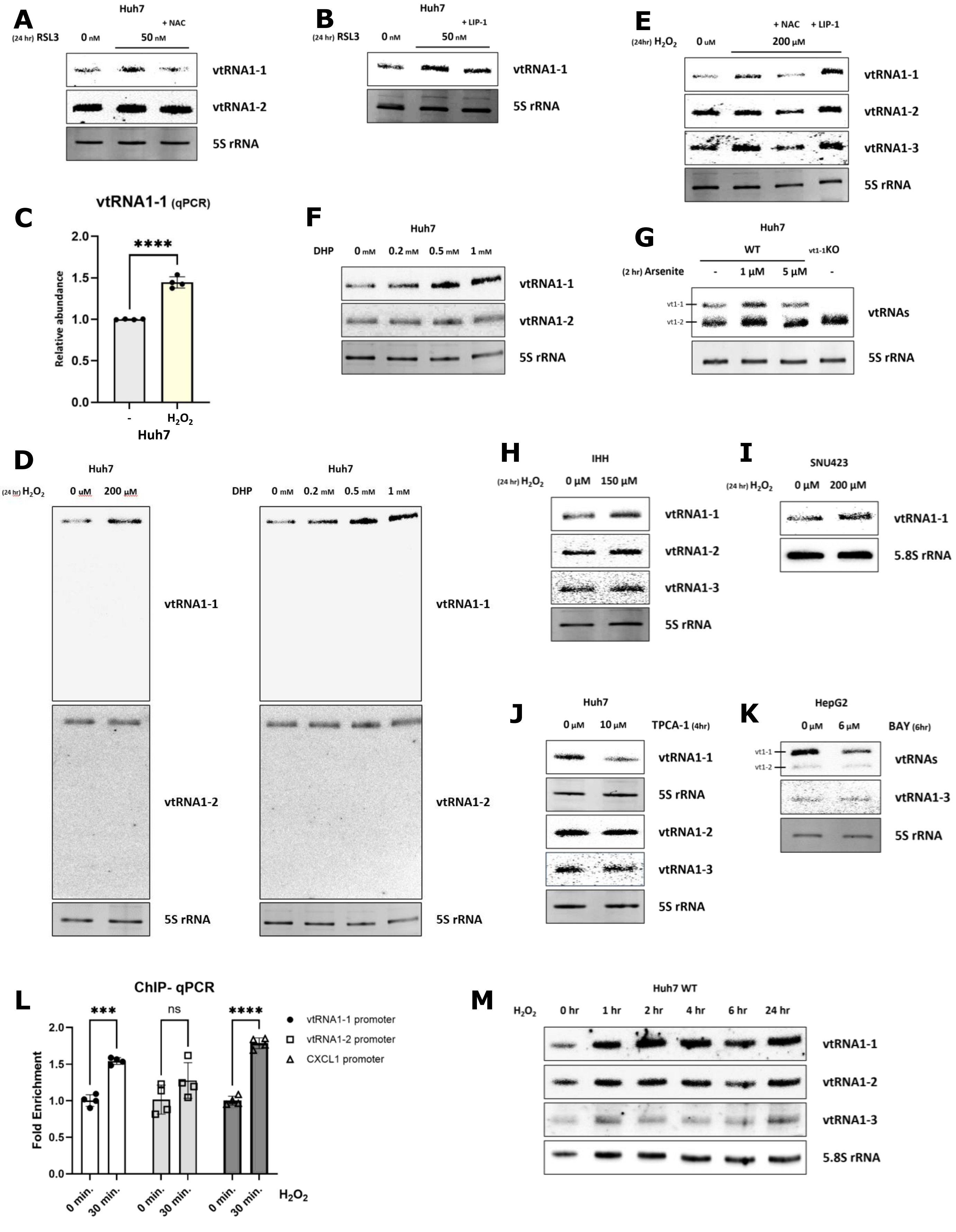
A
Huh7
(24 hr) RSL3
0 nM
50 nM
+ NAC
vtRNA1-1
vtRNA1-2
5S rRNA
B
Huh7
(24 hr) RSL3
0 nM
50 nM
+ LIP-1
vtRNA1-1
5S rRNA
E
(24hr) $H_2O_2$
0 uM
200 µM
+ NAC
+ LIP-1
vtRNA1-1
vtRNA1-2
vtRNA1-3
5S rRNA
C
vtRNA1-1 (qPCR)
****
Relative abundance
2.0
1.5
1.0
0.5
0.0
-
$H_2O_2$
Huh7
F
Huh7
DHP
0 mM
0.2 mM
0.5 mM
1 mM
vtRNA1-1
vtRNA1-2
5S rRNA
G
Huh7
WT
vt1-1KO
(2 hr) Arsenite
-
1 µM
5 µM
-
vt1-1
vt1-2
vtRNAs
5S rRNA
D
Huh7
(24 hr) $H_2O_2$
0 uM
200 µM
vtRNA1-1
vtRNA1-2
5S rRNA
Huh7
DHP
0 mM
0.2 mM
0.5 mM
1 mM
vtRNA1-1
vtRNA1-2
5S rRNA
H
IHH
(24 hr) $H_2O_2$
0 µM
150 µM
vtRNA1-1
vtRNA1-2
vtRNA1-3
5S rRNA
I
SNU423
(24 hr) $H_2O_2$
0 µM
200 µM
vtRNA1-1
5.8S rRNA
J
Huh7
0 µM
10 µM
TPCA-1 (4hr)
vtRNA1-1
5S rRNA
vtRNA1-2
vtRNA1-3
5S rRNA
K
HepG2
0 µM
6 µM
BAY (6hr)
vt1-1
vt1-2
vtRNAs
vtRNA1-3
5S rRNA
L
ChIP- qPCR
***
ns
****
Fold Enrichment
2.0
1.5
1.0
0.5
0.0
0 min.
30 min.
0 min.
30 min.
0 min.
30 min.
$H_2O_2$
vtRNA1-1 promoter
vtRNA1-2 promoter
CXCL1 promoter
M
Huh7 WT
$H_2O_2$
0 hr
1 hr
2 hr
4 hr
6 hr
24 hr
vtRNA1-1
vtRNA1-2
vtRNA1-3
5.8S rRNA

**Figure S5.** (A-B, D-K, and M) Northern blot analyses of vtRNAs. Total RNA was extracted from cells treated with indicated drugs and analyzed. 5.8S rRNA or 5S rRNA served as loading controls. Final concentrations were as follows: 5 mM NAC, 1 μM LIP-1, and 10 μM TPCA-1. (C) qPCR analysis of vtRNA1-1 in Huh7 WT cells treated $H_2O_2$ (n=4). ****$p<0.0001$ (t-test) (L) Chromatin immunoprecipitation using an NF-κB p65 antibody followed by qPCR analysis of the indicated gene promoters in Huh7 WT cells treated $H_2O_2$ for 30 minutes (n=4). The known NF- κB-target CXCL1 promoter served as positive control. ****$p<0.0001$, ***$p<0.001$, ns: non-significant (one-way ANOVA test)

# Figure S6

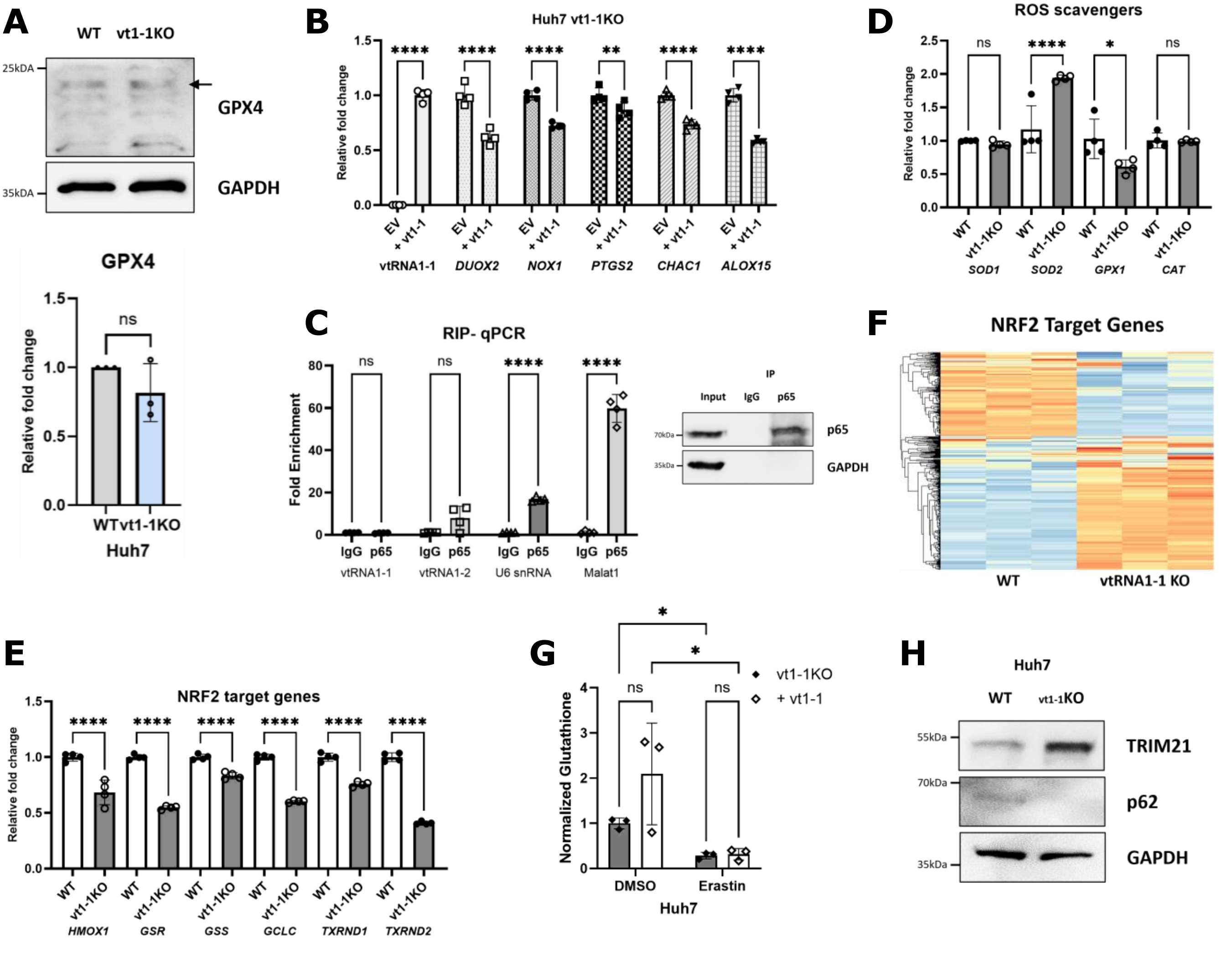
A
WT vt1-1KO
25kDA
35kDA
GPX4
GAPDH
GPX4
Relative fold change
ns
WT vt1-1KO
Huh7
B
Huh7 vt1-1KO
Relative fold change
EV
+ vt1-1
vtRNA1-1
DUOX2
NOX1
PTGS2
CHAC1
ALOX15
C
RIP- qPCR
Fold Enrichment
IgG p65
vtRNA1-1
vtRNA1-2
U6 snRNA
Malat1
IP
Input IgG p65
70kDa
35kDa
p65
GAPDH
D
ROS scavengers
Relative fold change
WT
vt1-1KO
SOD1
SOD2
GPX1
CAT
E
NRF2 target genes
Relative fold change
HMOX1
GSR
GSS
GCLC
TXRND1
TXRND2
F
NRF2 Target Genes
WT
vtRNA1-1 KO
G
Normalized Glutathione
vt1-1KO
+ vt1-1
DMSO
Erastin
Huh7
H
Huh7
WT vt1-1KO
55kDa
70kDa
35kDa
TRIM21
p62
GAPDH

**Figure S6.** (A and H) Immunoblot analysis using the indicated antibodies. Lysates were obtained from each cell and analyzed. GAPDH served as a loading control. The intensity of GPX4 was normalized to GAPDH (A, bottom, n=3). Error bars indicate the standard deviation. ns: non-significant (t test) (B, D, and E) qPCR analyses of the indicated mRNAs in control and vtRNA1-1-overexpressed Huh7 cells (B) or Huh7 WT and vtRNA1-1 KO cells (D and E) (n=4). EV denotes empty vector (mock transfection). (C) Native RNA immunoprecipitation (RIP) using an NF-κB p65 antibody followed by qPCR analysis of the indicated RNAs (F) A heatmap showing the expression levels of NRF2 target genes in Huh7 WT and vtRNA1-1 KO cells. ****$p<0.0001$, **$p<0.01$, ns: non-significant (two-way ANOVA test) (G) Indicated Huh7 cells were treated with Erastin for 16 hours, and total glutathione levels were measured.

# Figure S7

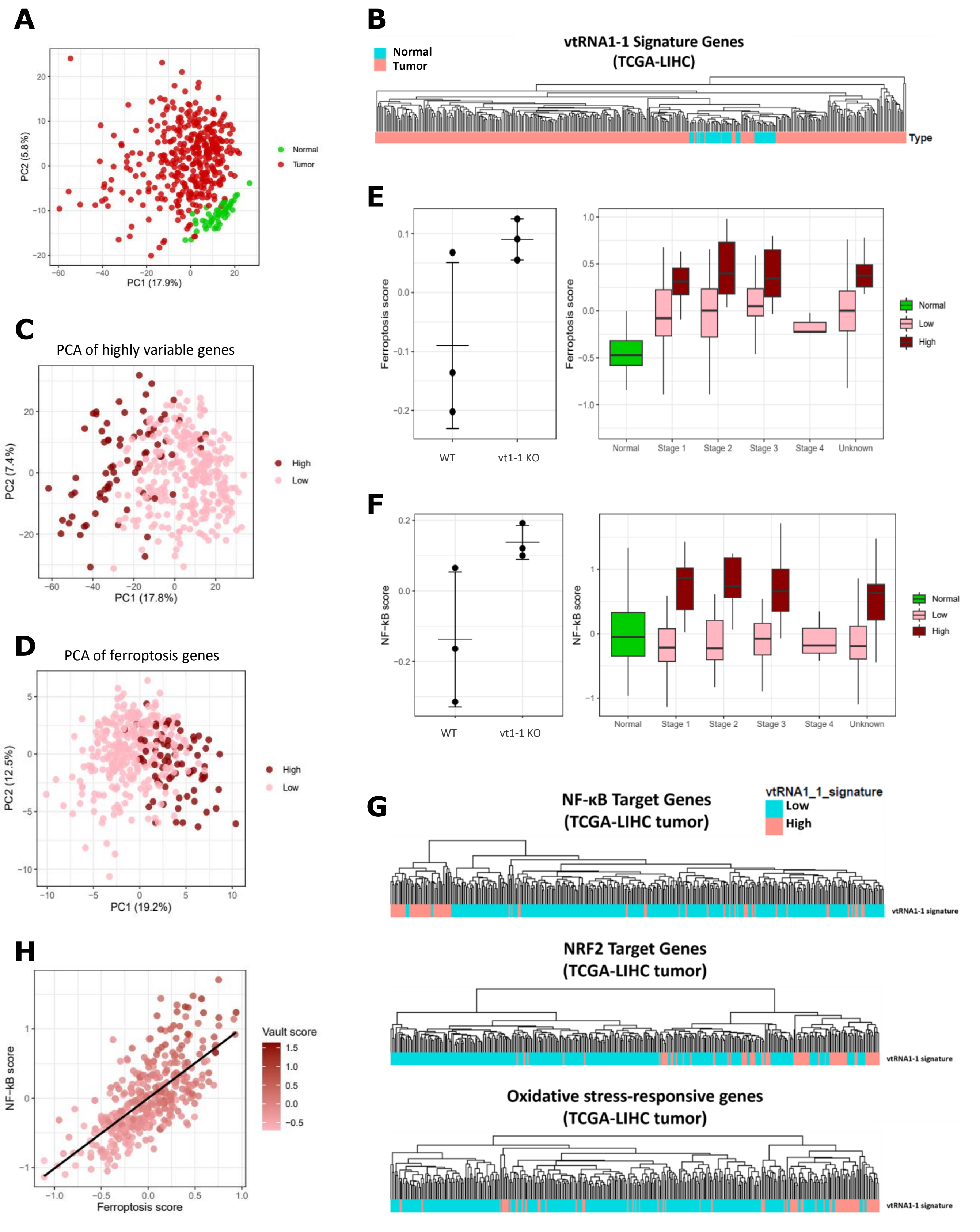
A
PC1 (17.9%)
PC2 (5.8%)
Normal
Tumor
B
vtRNA1-1 Signature Genes
(TCGA-LIHC)
Normal
Tumor
Type
C
PCA of highly variable genes
PC1 (17.8%)
PC2 (7.4%)
High
Low
D
PCA of ferroptosis genes
PC1 (19.2%)
PC2 (12.5%)
High
Low
E
Ferroptosis score
WT
vt1-1 KO
Normal
Stage 1
Stage 2
Stage 3
Stage 4
Unknown
Normal
Low
High
F
NF−κB score
WT
vt1-1 KO
Normal
Stage 1
Stage 2
Stage 3
Stage 4
Unknown
Normal
Low
High
G
NF-κB Target Genes
(TCGA-LIHC tumor)
vtRNA1_1_signature
Low
High
vtRNA1-1 signature
NRF2 Target Genes
(TCGA-LIHC tumor)
vtRNA1-1 signature
Oxidative stress-responsive genes
(TCGA-LIHC tumor)
vtRNA1-1 signature
H
NF−κB score
Ferroptosis score
Vault score

**Figure S7.** vtRNA1-1-associated expression signature aligns with transcriptional programs of ferroptosis-related genes in the TCGA-LIHC cohort. The clinical relevance of our findings by analyzing the TCGA-liver hepatocellular carcinoma (LIHC) cohort. To bypass the absence of vtRNA1-1 sequencing data in the TCGA database, we defined a vtRNA1-1-associated mRNA transcriptional signature using the 1,102 differentially expressed genes (DEGs) of vtRNA1-1 KO cells in our previous transcriptomic data [ref. 32 of the main text]. (A) Principal component analysis (PCA) plot based on the vtRNA1-1-associated signature genes showing entire normal (green) and tumor (red) samples across the TCGA-LIHC cohort. (B) Hierarchical clustering analysis based on the vtRNA1-1 signature genes across the TCGA-LIHC cohort. Blue and red represent the Normal and Tumor, respectively. These analyses reveal that tumor samples are clearly distinguishable from normal samples based on the vtRNA1-1 signature. (C and D) TCGA-LIHC tumor samples were divided into two groups based on the vtRNA1-1 signature score: a group exhibiting higher vtRNA1-1 score (High, top 20%) that expressed the vtRNA1-1 signature genes like vtRNA1-1 KO cells, and a group exhibiting lower vtRNA1-1 score (Low, bottom 80%) that expressed the signature genes like WT cells. PCA plot based on top 2,000 highly variable genes (C) and on the ferroptosis-related genes (D) across the TCGA-LIHC primary tumors. Dark red and pink dots represent the High and Low groups, respectively. These analyses indicate an association between the vtRNA1-1 signature and the transcriptional profile of ferroptosis-related genes. (E and F) Elevated expression of ferroptosis genes (ferroptosis scores) and NF-κB target genes (NF-κB scores) were observed in the transcriptomic data of vtRNA1-1 KO cells compared to WT cells**,** as well as in the High group of TCGA-LIHC cohorts regardless of tumor stages. Ferroptosis scores (E) and NF-κB scores (F) in transcriptomic data from WT and vtRNA1-1 KO Huh7 cells (left) and the TCGA-LIHC primary tumors (right). Green, dark red and pink represent the Normal, High, and Low groups, respectively. (G) Hierarchical clustering analysis based on the NF-κB target genes (top), the NRF2 target genes (middle) or the oxidative stress-responsive genes (bottom) across the TCGA-LIHC primary tumors. Red and blue represent the High and Low groups, respectively. (H) Positive relationship between ferroptosis and NF-κB scores in the TCGA-LIHC primary tumors, colored by vtRNA1-1 signature score.

**Probe sequences for northern blot**

| Probe (NB) | Sequence (5' - 3') |
|---|---|
| vtRNA1-1 | GCTTGTTTCAATTAAAGAACTGTCG |
| vtRNA1-2 | AGGTGGTTACAATGTACTCGAAG |
| vtRNA1-3 | GAGGTGGTTTGATGACACGCGAA |
| 5.8S rRNA | TCCTGCAATTCACATTAATTCTCGAGCTAGC |

**Primer sequences for RT-qPCR**

| Primer (qPCR) | Sequence (5' - 3') |
|---|---|
| vtRNA1-1 (Fw) | GGCTGGCTTTAGCTCAGC |
| vtRNA1-1 (Rv) | CCAGACAGGTTGCTTGTT |
| GAPDH (Fw) | TCAAGGCTGAGAACGGGAAG |
| GAPDH (Rv) | CGCCCCACTTGATTTTGGAG |
| 18S rRNA (Fw) | GAGAAACGGCTACCACATCCA |
| 18S rRNA (RV) | CTCCAATGGATCCTCGTTAAAGG |
| CHAC1 (Fw) | GGTGACGCTCCTTGAAGATC |
| CHAC1 (Rv) | CACTGCCTCTCGCACATT |
| ALOX15 (Fw) | CTTCCTAACCTACAGCTCCTTCT |
| ALOX15 (Rv) | ACAGCCACGTCTGTCTTATAGT |
| PTGS2 (Fw) | GATGATTGCCCGACTCCCTT |
| PTGS2 (Rv) | GGCCCTCGCTTATGATCTGT |
| DUOX2 (Fw) | ACGCAGCTCTGTGTCAAAGGT |
| DUOX2 (Rv) | TGATGAACGAGACTCGACAGC |
| NOX1 (Fw) | CAATCTCTCTCCTGGAATGGCATC |
| NOX1 (Rv) | CTGCTGCTCGGATATGAATGGAG |
| NOS2 (Fw) | TGCAGACACGTGCGTTACTC |
| NOS2 (Rv) | GGTAGCCAGCATAGCGGATG |
| XDH (Fw) | GACAGTTGTGGCTCTTGAGGT |
| XDH (Rv) | GGAAGGTTGGTTTTGCACAGC |
| BCL2L1 (Fw) | CGGTACCGGCGGGCATTCAG |
| BCL2L1 (Rv) | CGGCTCTCGGCTGCTGCATT |
| FSP1 (Fw) | CCCTGCCCTTCTCTCATCTTA |
| FSP1 (Rv) | TGTCCTCATAGGCCTGGATAG |
| SOD1 (Fw) | CCAGTGCAGGTCCTCACTTTA |
| SOD1 (Rv) | GTCTCCAACATGCCTCTCTTCA |
| SOD2 (Fw) | GGCTCCGGTTTTGGGGTATC |
| SOD2 (Rv) | GCATGATCTGCGCGTTGATG |
| GPX1 (Fw) | GTGTATGCCTTCTCGGCGC |
| GPX1 (Rv) | GACCGTGGTGCCTCAGAG |
| CAT (Fw) | CAGCGACCAGATGCAGCA |
| CAT (Rv) | CCACGGGGCCCTACTGTAA |
| HMOX1 (Fw) | AAGACTGCGTTCCTGCTCAA |
| HMOX1 (Rv) | GGGCAGAATCTTGCACTTTGT |

| | |
|---|---|
| GSR (Fw) | ATGGTCTGTGCTAACAAGGAAGA |
| GSR (Rv) | GTTGCTCCCATCTTCACTGCA |
| GSS (Fw) | GGGAGCCTCTTGCAGGATAAA |
| GSS (Rv) | GAATGGGGCATAGCTCACCAC |
| GCLC (Fw) | GGAGACCAGAGTATGGGAGTT |
| GCLC (Rv) | CCGGCGTTTTCGCATGTTG |
| TXRND1 (Fw) | TCACCCCAGTTGCAATCC |
| TXRND1 (Rv) | GTTGGAACATTTTCATAGTCACA |
| TXRND2 (Fw) | CGGCTTCGACCAGCAAATG |
| TXRND2 (Rv) | ACAGGACGGTGTCAAAGGTG |
| vtRNA1-1 P (Fw) | GGGGTTGGAATGTTTATCTAGTTTA |
| vtRNA1-1 P (Rv) | ACTTCGTGATCAAATCGACTTTTA |
| vtRNA1-2 P (Fw) | CTCTAACAAAAAAAATTTAAAAAGC |
| vtRNA1-2 P (Rv) | GCGTCTTTACAAGGTGTAAATTG |
| CXCL1 P (Fw) | ACTCGGGATCGATCTGGAACTC |
| CXCL1 P (Rv) | CTCCGAGATCCGCGAACCC |
